# A comprehensive proteomic analysis of elaioplasts from citrus fruits reveals insights into elaioplast biogenesis and function

**DOI:** 10.1038/s41438-017-0014-x

**Published:** 2018-02-07

**Authors:** Man Zhu, Jiajia Lin, Junli Ye, Rui Wang, Chao Yang, Jinli Gong, Yun Liu, Chongling Deng, Ping Liu, Chuanwu Chen, Yunjiang Cheng, Xiuxin Deng, Yunliu Zeng

**Affiliations:** 1 Key Laboratory of Horticultural Plant Biology (Ministry of Education), College of Horticulture and Forestry Science, Huazhong Agricultural University, Wuhan, 430070 China; 2 Institute of Citrus Science, Huazhong Agricultural University, Wuhan, 430070 China; 3 Shanghai Applied Protein Technology Co. Ltd, Shanghai, 200233 China; 4 Guangxi Citrus Research Institute, Guangxi, 541004 China

## Abstract

Elaioplasts of citrus peel are colorless plastids which accumulate significant amounts of terpenes. However, other functions of elaioplasts have not been fully characterized to date. Here, a LC–MS/MS shotgun technology was applied to identify the proteins from elaioplasts that were highly purified from young fruit peel of kumquat. A total of 655 putative plastid proteins were identified from elaioplasts according to sequence homology *in silico* and manual curation. Based on functional classification via Mapman, ~50% of the identified proteins fall into six categories, including protein metabolism, transport, and lipid metabolism. Of note, elaioplasts contained ATP synthase and ADP, ATP carrier proteins at high abundance, indicating important roles for ATP generation and transport in elaioplast biogenesis. Additionally, a comparison of proteins between citrus chromoplast and elaioplast proteomes suggest a high level of functional conservation. However, some distinctive protein profiles were also observed in both types of plastids notably for isoprene biosynthesis in elaioplasts, and carotenoid metabolism in chromoplasts. In conclusion, this comprehensive proteomic study provides new insights into the major metabolic pathways and unique characteristics of elaioplasts and chromoplasts in citrus fruit.

## Citrus fruits: The proteins of elaioplasts

A study of the proteins in bodies called elaioplasts in citrus fruit cells will help to clarify elaioplast formation and functions. Plant cells contain several types of membrane-bound bodies called plastids, with elaioplasts being an incompletely characterized type. They are known to store oils in the cells, especially molecules called terpenes that influence the fragrances and flavors of citrus fruits. Researchers in China, led by Yunliu Zeng at Huazhong Agricultural University, analyzed the protein molecules in elaioplasts of kumquat peel to investigate possible roles for elaioplasts other than oil storage. They identified more than 600 proteins and assigned likely functions to many. The results suggest the proteins are involved in chemical energy transactions and the manufacture of various categories of biomolecules. This will guide research to fully characterize the activities and significance of elaioplasts.

## Introduction

Plants contain several kinds of plastids, such as chloroplasts in photosynthetic tissues (e.g., leaves and green fruit), chromoplasts in pigment-containing fruits, as well as colorless leucoplasts in starch-storing or lipid-storing seeds^1^. These plastids are reported as the sites for several essential processes, including carbon fixation, nitrogen assimilation, and the biosynthesis of isoprenoids, starches, fatty acids (FA), and amino acids. Although plastids are a family of organelles possessing the same plastid genome (encoding approximately 100 proteins), the majority (>95%) of the 2000–3500 different proteins are imported from the surrounding cytoplasm rather than being synthesized *in situ*^2,3^. It is the imported proteins that define the developmental fate of plastids, leading to the different morphologies and special functions within specific plastids^4^. Recently, large-scale proteomics analysis has become an efficient approach for the investigating subcellular organelles at the protein level^2^. Special attention has been given to chloroplasts as well as chromoplasts in several species, including tomato, citrus, pepper, papaya, watermelon, carrot, and orange curd cauliflower^5–11^. In contrast, relatively few proteomics studies have focused on other types of plastid.

Leucoplasts are colorless plastids that include amyloplasts, elaioplasts, and proteoplasts that store starches, lipids, or proteins, respectively. Amyloplasts have been well studied and have been shown to be enriched in many storage tissues, including seeds, tubers/root vegetables, and fruits. In contrast, elaioplasts are much less studied in plants. Besides their role in storage, elaioplasts were also reported to possess crucial biosynthetic functions, for example in the biosynthesis of long-chain FAs in developing castor seeds^12^ and terpenes in the outer peel of citrus^13,14^. Plastid isolation is a prerequisite not only for assessing proteome composition, but also for understanding metabolic processes, enzyme localization, metabolite transporters, and biogenesis. Although protocols for the isolation of elaioplasts are available for some plant tissues, including petals of snapdragon flowers (*Antirrhinum majus*)^15^, developing seeds of mustard (*Brassica campestris*)^16,17^, and maize (*Zea mays*)^18^, as well as the outer peel of citrus (*Citrofortunella mitis*)^14^, the proteome profile of elaioplasts in these tissues is still unknown.

Citrus are one of the most important horticultural crops around the world, and have a unique anatomical structure, which consists of the pericarp (peel) and an edible endocarp (flesh). The endocarp accumulates large amounts of carotenoids within chromoplasts, which not only bring the red, yellow, and orange colors of fruit flesh, but also provide several important groups of nutrients. The pigmented region of pericarp contains active secretory cells that are characterized by numerous elaioplasts. Elaioplasts have been demonstrated to be involved in the biosynthesis of terpenes in essential oil, which are then exported into the secretory pocket, greatly affecting the aroma and taste of the fruit^14,19^. As the major component of essential oil, terpenes have been employed for manufacturing of perfumes and flavorings, and they are also used as antiseptic preservative agents to improve the safety and shelf-life of fruit during storage^20,21^. Although significant progress has been made in understanding chromoplast differentiation in the peel of citrus fruits^22,23^, little information is available about the regulatory mechanisms underlying elaioplast biogenesis.

In the present study, a comprehensive proteomic analysis using nLC-MS/MS-Q Exactive technology was used to identify proteins in elaioplasts isolated from the peel of kumquat by density gradient centrifugation. A total of 655 plastid protein candidates were detected that were predicted to participate in the general metabolism or unique metabolic pathways in elaioplasts, including flavor synthesis and fruit maturation. Overall, the present study provides an important insight into elaioplast biogenesis and formation of essential flavors.

## Materials and methods

### Plant materials

The outer peel of ~150 earlier-stage green fruits (0.5–0.8 cm in diameter) of ‘Rong An’ kumquat (*Fortunella margarita* Swingle) was used for elaioplast isolation. Fresh mature pulp of ‘Hong Anliu’ orange (*C. sinensis*) was used for chromoplast isolation. Both citrus cultivars were collected from Guangxi Institute of Citrus Research, located at Guilin city, Guangxi, China.

### Elaioplast and chromoplast purification

Elaioplasts were purified according to the procedure described by Michel et al.^14^ with minor modifications. Approximately 10 g of tissue was cut into pieces at 4 °C and then gently homogenized in pre-cooled SPBTK buffer (50 mM Tricine-NaOH pH 7.8, 5 mM KCl, 0.3 M sucrose, 0.5% (w/v) soluble polyvinylpyrrolidone 360, and 0.1% (w/v) bovine serum albumin). The homogenized sample was passed through two layers of Micracloth (Calbiochem). The pooled filtrate was centrifuged for 8 min at 5000 × *g*. The pellet was carefully re-suspended in SPBTK buffer at pH 7 and then centrifuged for 90 s at 800 × *g*. The resulting supernatant was centrifuged at 5000 × *g* for 10 min. The suspension containing most of the plastids was gently layered onto the top of a discontinuous gradient of 0.75, 0.92, 1.20, 1.50 M sucrose in 50 mM Tricine-NaOH (pH 6.5) and then centrifuged at 100,000 × *g* for 90 min in a Hitachi P40ST rotor. Four zones, designated as bands 1, 2, 3, 4 from the top to the bottom of the gradient, were clearly separated by sucrose gradient centrifugation. Each band was carefully recovered and examined under an optical microscope at ×100 magnification (Cover-018; Olympus, Tokyo). The purity of each fraction was tested by western blot analysis (described below). The elaioplast layer (band 1) was recovered. Chromoplast isolation from sweet orange was performed as described previously^10^. The final pellets containing elaioplast or chromoplast fractions were collected and flash-frozen in liquid nitrogen and kept at −80 °C. The plastid samples were prepared in two biological replicates.

### Western blot analysis

Polyclonal antibodies were diluted as appropriate against plastid Rubisco large subunit (RubcL; 53 kDa, 1:3000), cytosolic UDP-glucose pyrophosphorylase (UDPase; 51.6 kDa, 1:2000), vacuolar ATPase (V-ATPase; 26–31 kDa, 1:2000), and mitochondrial voltage-dependent anion-selective channel protein 1 (VDAC1; 29 kDa, 1:2000), as well as two photosynthesis-related antibodies (light-harvesting complex (Lhca1 and Lhca4), 1:5000) from Agrisera^®^. A total of 30 μg of proteins were used for immunoblot analysis as previously described^10^. At least two independent replicate experiments were performed for each immunoblot with similar result.

### Protein extraction and digestion in solution

Protein extraction was performed as previously described^9^. The filter-assisted sample preparation method was adapted for protein digestion. Briefly, 200 μg of proteins were mixed with 100 μl of 100 mM dithiothreitol (DTT), 150 mM Tris–HCl pH 8, 4% (w/v) SDS, incubated at 100 °C for 10 min, and then cooled to 25 °C. Next, UA buffer (150 mM Tris–HCl, pH 8.0, 8 M Urea) was used to remove detergent, DTT, and other low-molecular-mass substances by repeated ultrafiltrate (10 kDa; Microcon units), and then centrifuged for 30 min at 14,000 × *g*. The samples were washed using 100 μl of UA buffer followed by centrifugation for 30 min at 14,000 × *g*, and then incubated under darkness for 30 min by adding 100 μl of UAI buffer (8 M Urea, 150 mM Tris–HCl, pH 8.0, 50 mM iodoacetamide). Further washes (three times) with 100 μl of UA buffer followed by two washes using 100 μl of 50 mM NH_4_HCO_3_ (pH 8.5) were performed. Finally, the resulting proteins were digested with 4 μg of trypsin (Promega) overnight (16–18 h) at 37 °C. The concentration of peptide mix was estimated by UV absorbance at 280 nm as described previously^9^.

### Q Exactive-based LC–MS/MS analysis

nLC-MS/MS analysis was performed on an Easy-nLC system equipped with a Thermo Scientific Q Exactive mass spectrometer. 5 μg of peptide mixture was injected onto an Acclaim PepMap100 trap column (nanoViper C18, 100 μm×2 cm, Thermo Scientific) fitted with a C18-reversed phase analytical column (Easy Column, 75 μm×10 cm, 3 μm resin, Thermo Scientific) in mobile phase A (0.1% formic acid in water). Peptides were eluted with a linear 120-min gradient of mobile phase B (0.1% formic acid in 84% acetonitrile) at a flow rate of 0.3 μl/min. The separated MS data were obtained as described in ref. ^9^

MS/MS spectra were searched with the MaxQuant software package (version 1.1.0.36) against non-redundant protein databases of *Citrus sinensis* (http://www.pytozome.com or http://citrus.hzau.edu.cn), and the complete chloroplast genome sequence of *C. sinensis* (accession no. NC_008334). The search parameters were performed as described in ref. ^9^ Only the proteins identified by two or more peptides in two bio-replicates were retained for further analysis.

### Functional categorization

Detected proteins were categorized based on Mapman functional bins (https://mapman.gabipd.org/mapman). Additional curation was performed according to classifications in PPDB (http://ppdb.tc.cornell.edu). The proteins identified in citrus plastids were queried against three comprehensive plastid proteome databanks (AT_CHLORO, http://www.grenoble.prabi.fr/at_chloro/; PPDB (Plastid Proteome Database, http://ppdb.tc.cornell.edu/); Plprot (Plastid Protein Database, http://www.plprot.ethz.ch/); downloaded in October 2016); and two plastidial protein subset databanks (Uniprot, http://www.uniprot.org/; SUBA, http://suba.plantenergy.uwa.edu.au/; downloaded in October 2016). There were 2816 plastid proteins in plprot, 1265 AT-CHLORO, 2292 in PPDB, 2977 in SUBA and 2038 in Uniprot.

Subcellular localization analysis was carried out using three software programs: Predotar (https://urgi.versailles.inra.fr/predotar/), Target P (http://www.cbs.dtu.dk/services/TargetP/), and WoLF PSORT (http://www.genscript.com/wolf-psort.html). Proteins were tentatively assigned as plastidial (i) if predicted to be plastid targeted by at least one of the three programs, and/or (ii) if homologous to plastid proteins annotated in at least two plastidial databanks. Additionally, sequence information was manually curated if they were available in the literature. The putative plastid proteins of elaioplast and chromoplast were listed in Supplementary Table S1.

### Microscopy observation and analysis

Isolated plastids were carefully layered onto microscope slides and visualized using a DP70 camera coupled to an Olympus BX61 microscope. Plastid samples for transmission electron microscopy (TEM) analysis were prepared and examined as described previously^9^. The plastid diameter was calculated by (minor axis + major axis)/2 with ImageJ (http://rsbweb.nih.gov/ij/).

## Results and discussion

### Isolation of highly purified elaioplasts from kumquat peel with density gradient centrifugations

**Fig. 1:**
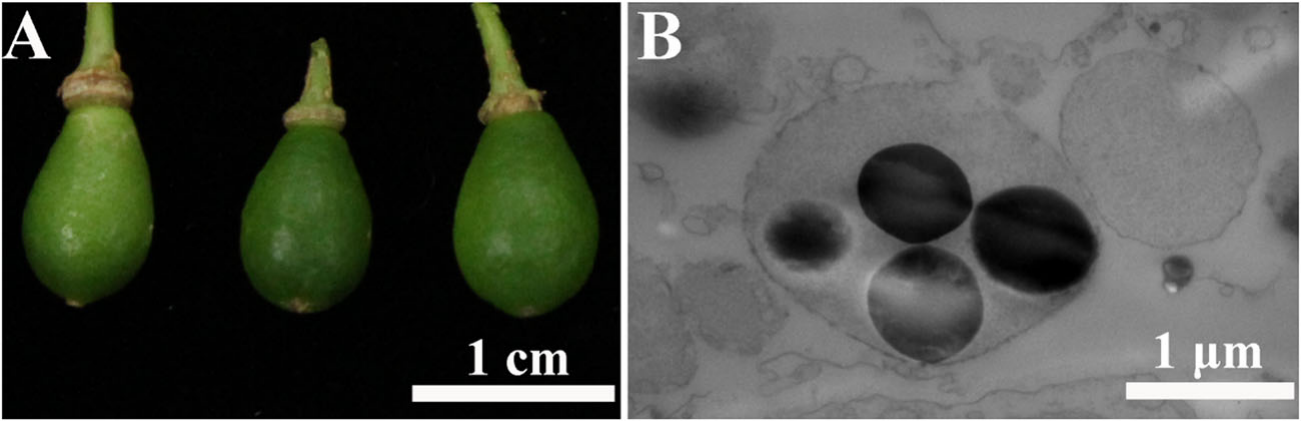
Fruits of kumquat and elaioplasts in the outer peel. **a** A representative photograph of kumquat fruits used for elaioplasts isolation. **b** Transmission electron microscopic (TEM) picture showing the ultrastructural features of elaioplasts in the outer peel of kumquat

Kumquat, a major citrus cultivar in southern China, bears the smallest fruits in the *Citrus* genus. Ripened fruits can be eaten as a whole (Fig. 1a), and elaioplasts with several plastoglobules were frequently found in the outer peel (Fig. 1b).

**Fig. 2:**
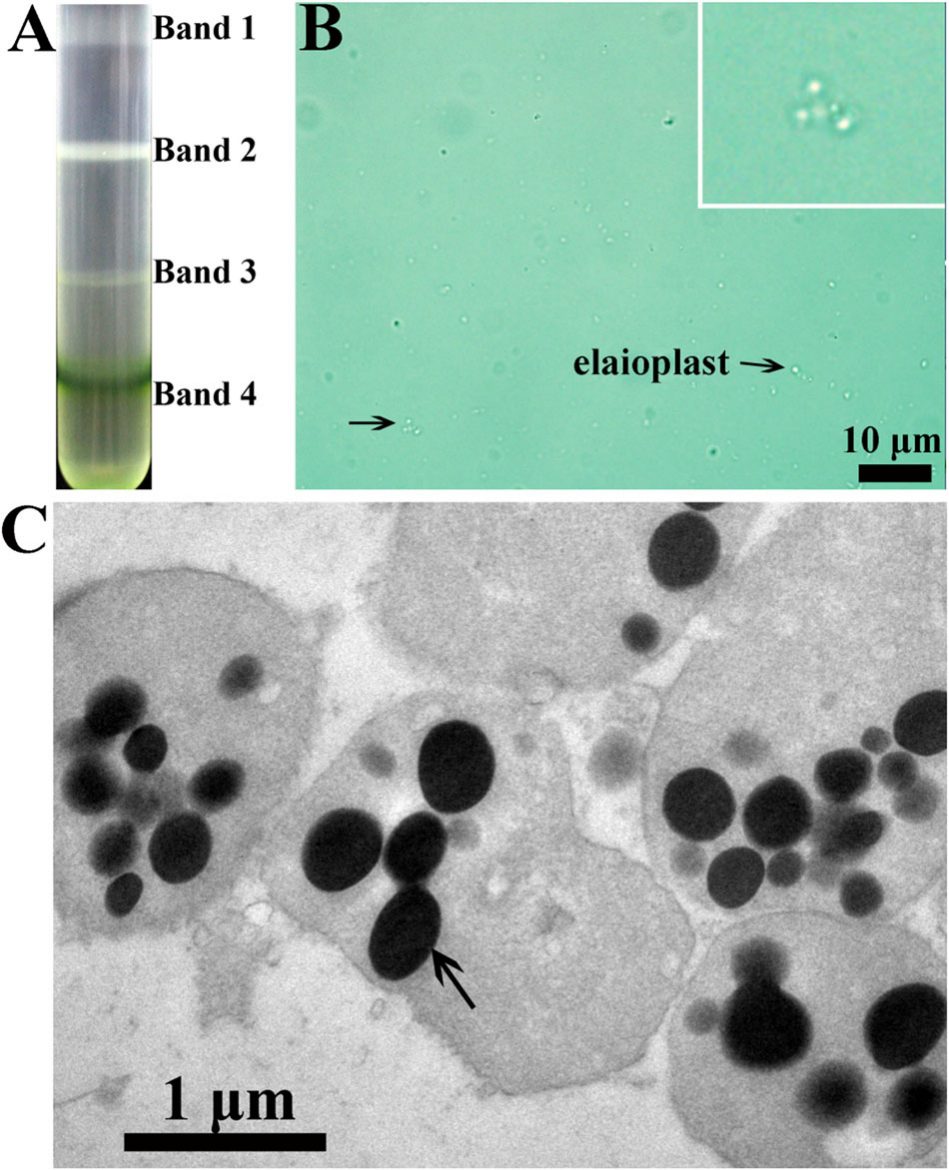
Isolation and ultrastructure of citrus peel elaioplasts. **a** Separation of elaioplasts on a discontinuous sucrose gradient (0.75, 0.92, 1.20, 1.50 M, Bands 1–4). **b** Light microscopy of purified elaioplasts. A representative micrograph of band 1 by density gradient contains a clean fraction of elaioplasts. Arrows indicate abundant colorless plastids with several sphere substructures clustering together. **c** An electron micrograph showing the ultrastructure of elaioplasts. The arrow indicates a plastoglobule

In this study, we initially isolated elaioplasts from the outer peel of kumquat. The recovered pellet containing elaioplasts was separated into four fractions on a discontinuous gradient comprising 0.75, 0.92, 1.20, 1.50 M sucrose (Fig. 2a). The elaioplasts pooled from band 1 were characterized by abundant colorless plastids with several sphere substructures clustering together, and their sizes ranged from 1 to 4 μm as measured under light microscopy and TEM (Fig. 2b, c). Analysis of the other bands indicated they were contaminated by either cell debris with few elaioplasts, or chloroplasts (Supplementary Figure S1).

**Fig. 3:**
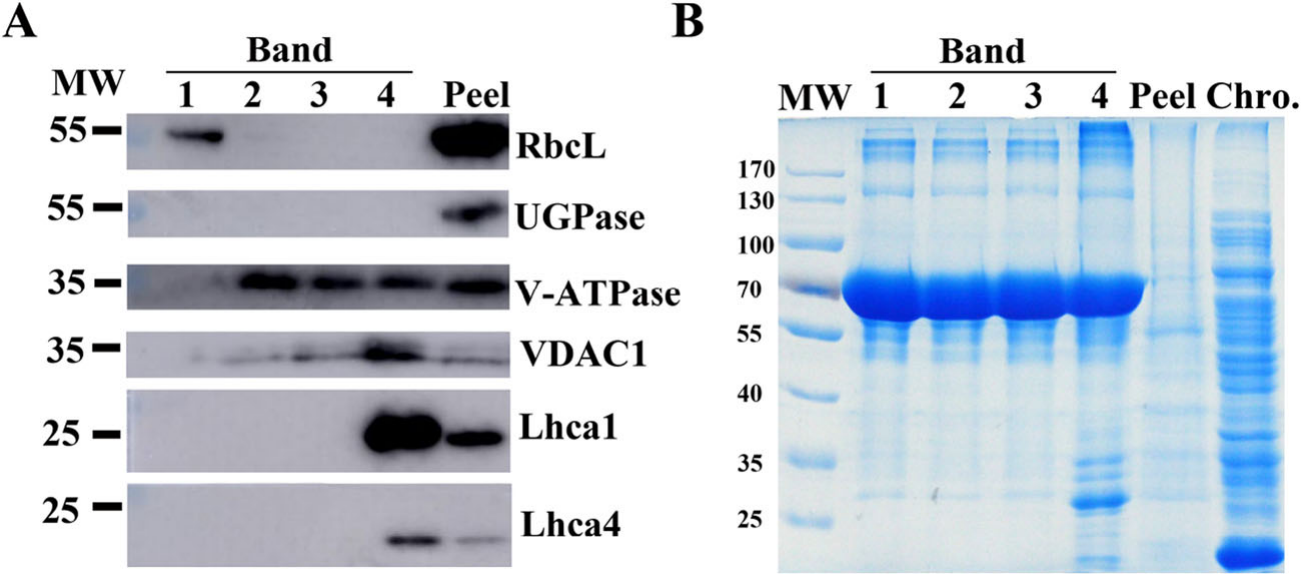
Assessment of the purity of isolated elaioplasts using immunoblots. Different fractions of the sucrose gradient (Band 1 to Band 4) are compared to peel proteins using antibodies for plastid stroma large Rubisco subunit (RbcL), cytosolic UGPase, vacuolar (v)-ATPase, mitochondrial voltage-dependent anion-selective channel protein 1 (VDAC1), and photosynthesis Light-harvesting complex (Lhca1 and Lhca4); **b** Coomassie blue protein profiles of purified elaioplasts (Band 1 to Band 4) from kumquat peel, as compared with purified chromoplast (Chro.) from sweet orange flesh and total kumquat peel proteins

The purity of the elaioplast-enriched protein fractions was monitored by immunoblot analysis with antibodies against marker proteins for the plastid stroma (RbcL), cytosol (UDPase), vacuole (V-ATPase), and mitochondrion (VDAC1), as well as chloroplast (Lhca1 and Lhca4). As shown in Fig. 3, proteins extracted from the purified elaioplast fraction readily reacted with the antibody of RbcL. By contrast, no reaction could be detected with the cytosolic UGPase, mitochondrial VDAC1, or vacuolar V-ATPase. Additionally, no reaction was detected with antibodies to PSI type I chlorophyll a/b-binding proteins (Lhca1 and Lhca4) involved in photosynthesis within the chloroplasts. Reactions with these antibodies were observed in the total proteins extracted from the kumquat peel which contained both chloroplasts and elaioplasts. The above results suggest that the isolated elaioplasts used for the subsequent proteome analysis were of high purity without contamination by mitochondria, cytosol, vacuole, and chloroplasts.

### An inventory of proteins in elaioplasts by functional classification and subcellular localization

**Fig. 4:**
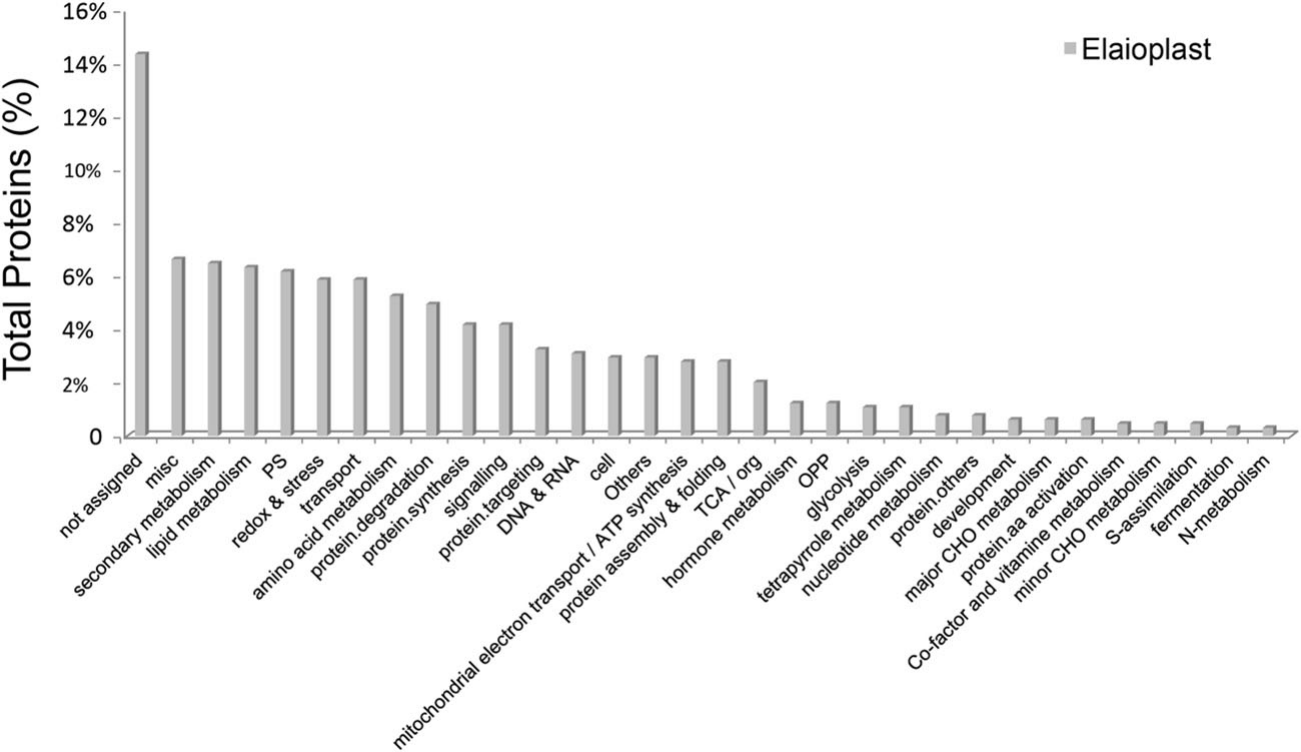
Functional categories of proteins identified from elaioplasts. Proteins were categorized into 32 functional classifications according to MapMan (verified by PPDB, https://mapman.gabipd.org/mapman)

We conducted a comprehensive proteomic analysis to characterize the proteins found in elaioplasts. A total of 1239 proteins with at least two detected peptides in two bio-replicates, were identified from purified elaioplasts. The raw data obtained from the elaioplast proteins were refined by searches against five plastid databanks (Plprot, AT-CHLORO, SUBA, PPDB, and Uniprot) and by three targeting predictors (WoLF PSORT, Predotar, and Target P). Based on the chosen criteria for plastid-localized proteins (Materials and Methods), an inventory of 655 putative plastid proteins was constructed, accounting for 52% of all identified proteins (Supplementary Table S1). Among them, 562 proteins (accounting for 85.8% of these identified proteins) were predicted to be plastid targeted by at least one predictor program. WoLF PSORT, Target P, and Predotar predicted plastid-localization of 76.0% (498 proteins), 38.2% (250 proteins), and 31.9% (209 proteins) of the 655 identified proteins, respectively. Two hundred and twenty-nine proteins (35.0%) were predicted to be plastid targeted by at least two predictors, suggesting that these proteins are highly likely to be plastid localized. As shown in Supplementary Table S1 and in Fig. 4, the 655 putative elaioplast proteins were categorized into 32 functional classes according to MapMan bins.

### Major metabolic functions in the citrus elaioplast proteome

The identification of proteome profiles allows an overview of proteins involved in various metabolic pathways^8,24–28^. As shown in Fig. 4, the major metabolic pathways in citrus elaioplasts are protein metabolism and process, transport, secondary metabolism, lipid metabolism, redox and stress, amino acid metabolism, photosynthesis, DNA and RNA, and miscellaneous.

Elaioplasts are a type of heterotrophic plastid, wherein all the metabolic precursors are either generated via oxidative metabolism or actively imported from the cytosol to sustain anabolic reactions^29^. The proteins of the Calvin cycle are involved in providing intermediates for glycolysis, and proteins of the oxidative pentose phosphate pathway (OPPP) serve as a major source of reducing power for various metabolic pathways. As expected, we identified several proteins involved in the Calvin cycle within elaioplasts, such as fructose-bisphosphate aldolase, phosphoglycerate kinase, and transketolase. Furthermore, proteins participated in oxidative metabolism were well presented, including glucose-6-phosphate dehydrogenase, 6-phosphogluconolactonase, and 6-phosphogluconate dehydrogenase. Of note, transketolase and transaldolase (key enzymes of the non-oxidative part of OPPP), which are reported to feed the processes of anabolism or glycolysis^30^, were also identified in the present study. Glycolysis also occurs in plastids to generate ATP and pyruvate in plants^31^. Several glycolysis enzymes including aldolase, phosphoglycerate kinase, enolase, and pyruvate kinase were observed. Consistent with previous findings in non-photosynthetic plastids, a substantial number of proteins responsible for the generation of ATP and reducing power were observed^8^, indicating their high conservation among different plastid types. Taken together, the observation of many proteins involved in essential biosynthetic activities suggests an active metabolism in elaioplasts to produce reducing power and energy, as well as the supply of precursors for the biosynthesis of metabolites.

Several proteins involved in photosynthesis were found in elaioplasts although the presence of chloroplasts was not observed using light microscopy and there was no signal obtained by immunoblotting using two photosynthesis-related antibodies (Fig. 3a). This suggests that these proteins serve roles in processes other than photosynthesis in elaioplasts. It is no surprise to find photosynthetic proteins in elaioplasts, since similar observations have been made in other types of non-photosynthetic plastids, such as chromoplasts^6,10^ and amyloplasts^26^.

FA are known to be synthesized in plastids^28^. We detected several enzymes for the synthesis of FA as well as glycolipids and phospholipids in elaioplasts, such as acetyl E1 beta pyruvate dehydrogenase complex, E2-dihydrolipoamide acetyltransferase, E3-dihydrolipoamide dehydrogenase 1, long-chain acyl-CoA synthetase 7/8/9, and plastidial ketoacyl-ACP reductase (Supplementary Table S1). The accumulated FA could be converted into phosphatidic acid and/or imported into plastids via the trigalactosyldiacylglycerol (TDG) complex transporter, which is targeted to the plastid envelope membrane^32^. As expected, several key enzymes involved in lipid metabolism and homeostasis were detected in elaioplasts, such as TDG2, phosphatidylglycerolphosphate synthase 1, sulfoquinovosyldiacylglycerol synthase, as well as 3-ketoacyl-CoA thiolase, enoyl-CoA hydratase, phospholipase Dα1, and cyclopropane-fatty-acyl-phospholipid synthase. These results indicate that the elaioplasts have the ability to synthesize FAs and polar lipids that might relate to the formation of new membranes during elaioplast development^33^. This finding is also in agreement with a recent metabolic analysis in isolated tomato fruit chromoplasts using radiolabeled precursors^34^, which suggests a highly conservative mechanism for lipid biosynthesis and homeostasis in both chromoplasts and elaioplasts. It has been well documented that lipoxygenases (LOX), which are associated with generation of polyunsaturated FA hydroperoxides, could contribute to the biosynthesis of jasmonic acid (JA)^35^ and FA-derived flavor compounds^36^. In the present study, several enzymes involved in the LOX pathway (such as LOX1, LOX2, and LOX6) and JA metabolism (allene oxide cyclase 4) were detected. Therefore, it is possible that the presence of lipoxygenases in citrus elaioplasts and chromoplasts^10^ may play a key role in generating odor or flavor through production of LOX-derived volatiles in the outer peel and flesh of a fruit, respectively. A similar role for lipoxygenases in chromoplasts of tomato has been proposed^6^.

Elaioplasts are also important sites for amino acids synthesis. A large number of proteins responsible for amino acid metabolism were identified in elaioplasts, such as threonine synthase, aspartate aminotransferase, alanine transaminase, argininosuccinate synthase, cystathionine beta-lyase, 3-dehydroquinate synthase, isopropylmalate isomerase, isopropylmalate dehydrogenase, and ketol-acid reductoisomerase (Supplementary Table S1). Consistently, proteins of amino acid metabolism were also extensively found in non-photosynthetic plastids^8,10,26^. Taken together, our data suggest that these proteins involved in the biosynthesis of several types of amino acids, including aromatic amino acids have important functions in the active synthesis of amino acids within the elaioplasts.

Active redox system is a key regulator for plant development and fruit senescence and has a wide range of functions in gene expression, enzyme activities, and protein import in plastids^37^. In the elaioplast proteome, proteins involved in redox & stress constituted one of the top five functional classes. Several essential components in maintaining homeostasis of cell redox were found in the elaioplast proteome. These include the proteins involved in ascorbate-glutathione (AsA-GSH) cycle and antioxidant system of plastids, such as glutathione peroxidases, peroxiredoxins (PRXs), superoxide dismutases (SODs), and thioredoxin. Additionally, several key enzymes involved in second active oxygen species scavenging system were identified in elaioplasts, including the AsA-GSH cycle catalyzed by ascorbate peroxidases (APXs), as well as the AsA-dependent generation systems catalyzed by (momo)dehydroascorbate reductase^38^. Like in photosynthetic chloroplasts, APXs encountered in elaioplasts may indicate the presence of the AsA-dependent system involves the reduction of H_2_O_2_ using AsA as reductant^39^. The activation of plastid SOD expression was detected to be dependent on a copper chaperone for SOD^40^. Thus, as expected, Fe-SOD and Mn-SOD were detected and their overexpression induced an enhancement of plant tolerance in response to environmental stresses^41,42^. The presence of a large number of enzymes involved in oxidative stress response indicates a functionally active redox system in citrus elaioplasts and suggests the roles of these proteins in ROS metabolism, allowing the plastid to adapt to various environmental signals and processes during elaioplast development as fruit maturation proceeds.

Protein synthesis, degradation, and translocation regulate the biogenesis of plastids. In this study, proteins associated with import, ribosome assembly and proteases account for 16.9% of the total plastid proteins (Fig. 4). (1) Import of nuclear-encoded precursor proteins into plastids is mediated by translocon complexes at the outer and inner envelope membrane of chloroplasts (TOC/TIC complexes)^43^, which is important for the biogenesis of either chloroplasts^44^ or chromoplasts^1^. However, the import machinery remains unclear in elaioplasts. In this study, numerous translocons including TOC33/75/159 and TIC62/55/40/22/110 in the protein import machinery were identified in elaioplast proteome (Supplementary Table S1). Among the TOC complex members, TOC75, which is deeply embedded within outer membrane of plastid, plays a major role to form protein translocation channel^43^. TIC110, the major translocon component for protein translocation, plays a critical role in protein import into plastids^45^. The extensive identification of TOC/TIC complex likely plays an important role in elaioplast biogenesis on basis of protein import machinery. (2) Thirty ribosomal proteins of translation machinery were identified in citrus elaioplasts. These proteins are represented by plastid-encoded 30S and 50S subunit, together with nucleus-encoded 40S and 60S subunit. These findings suggest the presence of translation machinery in elaioplast biogenesis. (3) Maintaining a balance between protein synthesis, import, turnover, and processing is an important factor determining plastid fate and maintaining their homeostasis^1,46^. As expected, we identified numerous caseinolytic peptidase (Clp) proteases in the study (Supplementary Table S1), which have been well reported to form a chaperone complex to drive protein import, processing, and recycle plastid components. The knock-out of most members of Arabidopsis Clp proteases resulted in variegated/pale-green leaf phenotype, indicating their crucial roles in chloroplast development^47^. Elaioplast proteome also consists of several membrane-bound ATP-dependent metalloproteases (FtsH2, FtsH4, FtsH5, FtsH9, and FtsHi4), which have been described as critical for plastid development^48^. The extensive presence of Clp protease family in elaioplasts might contribute to elaioplast biogenesis by processing and/or cleavage of transit peptides of proteins in the plastids.

### Abundant proteins in citrus elaioplasts

The protein abundance for all proteins detected in kumquat elaioplasts was determined based on intensity-based absolute quantification (iBAQ). The 30 most abundant proteins of kumquat elaioplasts are listed in Table 1. Among them, we identified four enzymes involving in secondary metabolism. Three enzymes (i.e. DXS, HDS, and GGPS) belong to the 2-C-methyl-d-erythritol 4-phosphate (MEP) pathway, which provides precursors to synthesize monoterpenes within plastids. The fourth (orange1.1t03905) was a putative terpene synthase, which might be responsible for the accumulation of d-limonene (accounting for up to 90% among monoterpene compounds) in outer peel of citrus^49,50^. The importance of DXS in regulating MEP substrate flux in kiwifruit and grape has previously been reported^51,52^ and the identification of MEP pathway proteins and TPS genes in citrus elaioplasts indicates the key role that elaioplasts play in basic metabolism.

**Table 1:** Thirty most abundant proteins identified from elaioplasts

Accession	Rep. 1	Rep. 2	Protein description	Mapman	iBAQ in Rep. 1	iBAQ in Rep. 2	Average iBAQ
Cs7g01840	13	11	ADP, ATP carrier protein 2	Transport	27.7	27.4	27.6
Cs1g10270	27	28	Obsolete gene model variant (ATPase 70 kDa)	Transport	27.5	27.4	27.4
Cs3g24640	5	7	OEP24-II	Transport	27.0	26.8	26.9
orange1.1t02891	4	4	HP30-2 (PRAT family)	Transport	26.7	26.7	26.7
Cs2g14350	12	12	Phosphate transporter (PHT3-1 or PIC1)	Transport	26.6	26.7	26.7
Cs6g15300	42	44	ATP-binding cassette transporter	Transport	26.7	26.6	26.6
Cs2g13550	24	25	H^+^-transporting ATP synthase beta chain	Electron transport/ATP synthesis	28.6	28.7	28.6
orange1.1t01329	2	2	ATP synthase epsilon chain	Electron transport/ATP synthesis	27.5	27.4	27.4
Cs1g20530	15	15	1-deoxy-d-xylulose 5-phosphate synthase (DXS1)	Secondary metabolism	27.7	27.9	27.8
orange1.1t03905	1	1	(R)-limonene synthase 1	Secondary metabolism	27.9	27.5	27.7
Cs6g17510	11	11	Geranylgeranyl pyrophosphate synthase (GGPS1)	Secondary metabolism	27.5	27.4	27.5
Cs8g16700	29	28	4-hydroxy-3-methylbutyl diphosphate synthase (HDS)	Secondary metabolism	26.8	26.8	26.8
Cs5g33860	9	10	Thioredoxin family protein	Redox and stress	27.0	27.2	27.1
Cs7g16410	7	7	NADH-cytochrome b5 reductase	Redox and stress	26.6	26.5	26.5
Cs6g15550	8	6	Peroxiredoxin IIE (PrxII E)	Redox and stress	26.6	26.4	26.5
gi|113952630	17	19	Ribulose-1,5-bisphosphate carboxylase/oxygenase large subunit	PS	29.3	29.6	29.4
Cs2g03780	6	6	LHCII-1.2	PS	29.3	29.5	29.4
Cs6g18680	9	9	Rubisco small subunit 3b	PS	28.4	28.4	28.4
orange1.1t00226	15	14	Transketolase-2 (TKL-2)	PS	26.8	26.6	26.7
Cs6g14330	6	8	40 S ribosomal protein S14 (RPS14C)	Protein synthesis	27.4	27.3	27.4
Cs5g16780	8	10	Kunitz-type protease inhibitor	Protein degradation	27.0	27.1	27.1
orange1.1t01459	31	31	Cpn60 subunits	Protein assembly & folding	27.4	27.5	27.4
Cs7g29010	20	24	HSP70-1	Protein assembly & folding	26.7	26.8	26.7
Cs2g28210	13	15	Formate dehydrogenase	Others	27.7	27.6	27.7
Cs3g27530	3	2	Hp17	Not assigned	27.1	27.0	27.1
Cs2g21000	17	14	Hydroperoxide lyase1	Not assigned	26.9	27.0	27.0
Cs2g02520	13	14	Fibrillin 1b (FBN1b)	Misc	27.1	27.1	27.1
orange1.1t04376	31	35	Lipoxygenase AtLOX2, plastid	Hormone metabolism	27.9	27.7	27.8
Cs8g03410	9	11	Branched-chain amino acid transaminase 2	Amino acid metabolism	26.4	26.5	26.4
Cs9g14610	9	8	BCL-2-associated athanogene 7 (ATBAG7)	Signaling	27.4	27.3	27.4

Eight proteins involved in transport or electron transport categories, such as ADP, ATP carrier protein, ATP-binding cassette transporter, ATPase, as well as ATP synthase beta/epsilon chain were also detected in kumquat elaioplasts. ATPase acts as an essential regulator of membrane trafficking, which couples free energy generated from ATP hydrolysis to transport of substances across the membranes of many organelles^53^. ATP synthases are involved in energy production and proton transport. The identification of these proteins at high abundance suggests they may play an important role in active energy production and transport during elaioplast development. The presence of an active ATP generation system that is also necessary for the formation of membranes in plastids^54^. Elevated energy production has also be observed in other heterotrophic plastids, such as chromoplasts^6^ and proplastids^55^.

### The citrus elaioplast proteome compared with citrus chromoplast proteome

**Fig. 5:**
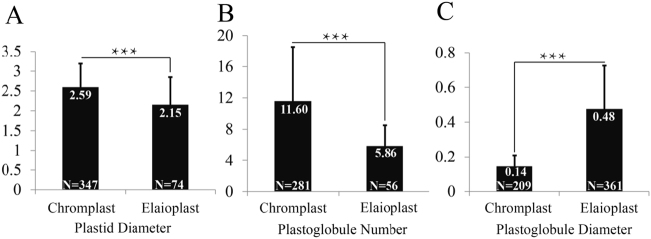
Ultrastructural differences between elaioplasts and chromoplasts. **a** The size of elaioplasts and chromoplasts in diameter. **b** The number of plastoglobules versus that of chromoplasts. **c** The size of plastoglobule of elaioplasts versus that of chromoplasts. Data are indicated as means ± SD and *p* values were calculated using Student’s *t* test in Microsoft Excel. ***p < 0.001; number (*N*) in the bars represents the number of plastids measured by using ImageJ software

To determine whether the ultrastructure was different between elaioplasts and chromoplasts, we purified chromoplasts from citrus flesh for comparison with elaioplasts. TEM analysis showed that the size of elaioplasts was 2.15 ± 0.70 μm in diameter, which was significantly smaller than that of chromoplast (2.59 ± 0.60 μm) (Fig. 5a; Supplementary Figure S2). The number of plastoglobules (5.86 ± 2.65) per elaioplast was approximately half that per chromoplast (11.59 ± 6.94) (Fig. 5b). However, the size of plastoglobules within elaioplasts was 0.48 ± 0.25 μm in diameter, which was about threefold greater than the diameter of plastoglobules within a chromoplast (0.14 ± 0.06 μm) (Fig. 5c). Taken together, the above results suggest that there are differences in ultrastructure between elaioplasts and chromoplasts.

**Fig. 6:**
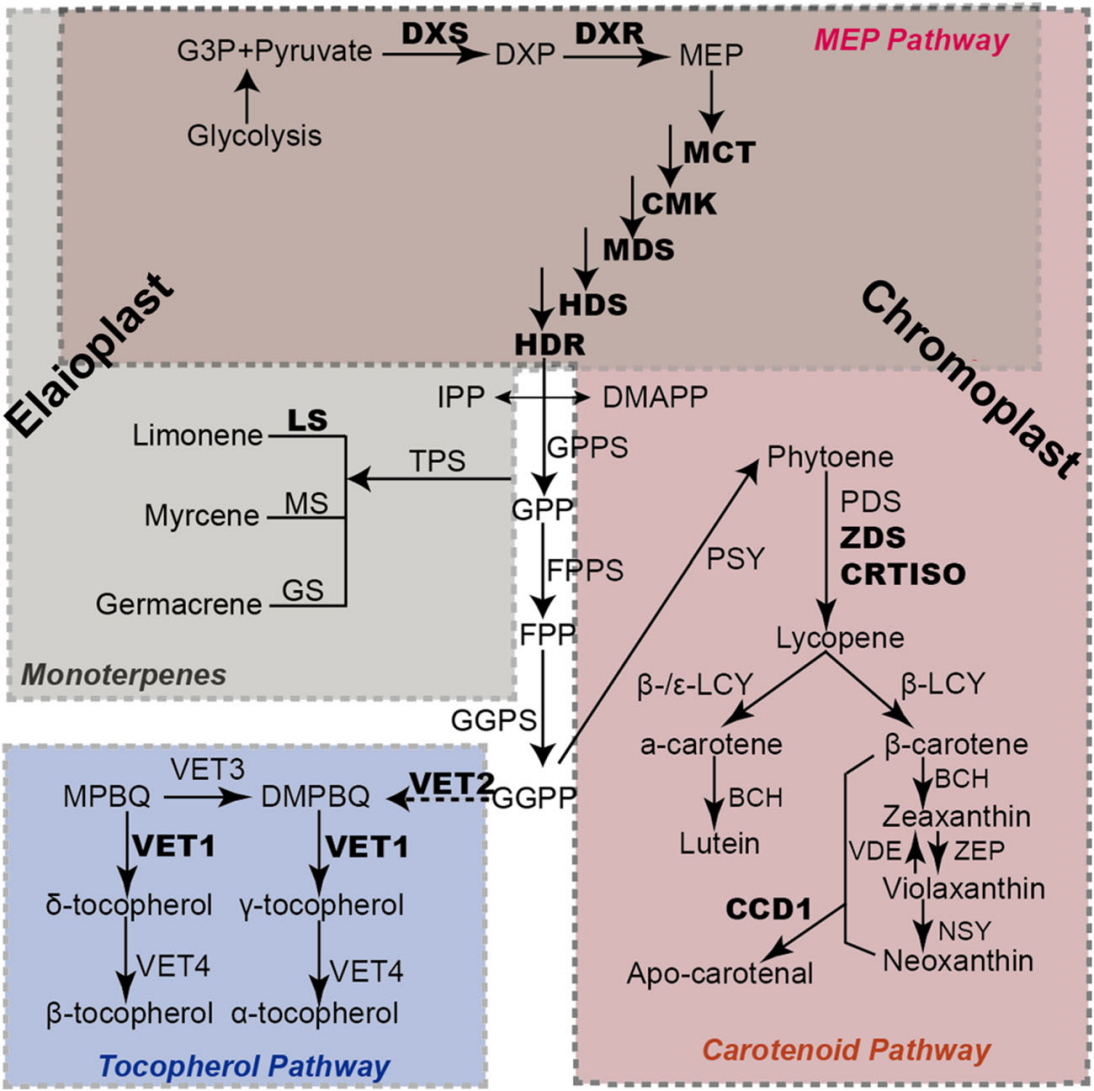
Schematic illustration of the biosynthesis of secondary metabolites in elaioplasts and chromoplasts. Proteins in boldface were identified in the proteomes of both plastid types. The full name of enzymes was listed in Supplementary Table S2. DXP 1-deoxy-d-xylulose 5-phosphate, MEP 2C-methylerythritol 4-phosphate, IPP isopentenyl diphosphate, DMAPP dimethylallyl pyrophosphate, GPP geranyl diphosphate, FPP farnesyl disphosphate, GGPP geranylgeranyl diphosphate, MPBQ 2-Methyl-6-phytyl-1,4-hydroquinol, DMPBQ 2,3-Dimethyl-5-phytyl-1,4-hydroquinol

Given the structural differences between elaioplasts and chromoplasts, it was not surprising that differences in protein distribution patterns on SDS-PAGE gels were also observed (Fig. 3b). We further explored these differences by comparing the elaioplast proteome with that of chromoplast at the mass spectrometric level (Supplementary Figure S3). Distinct proteome profiles for the biosynthesis and accumulation of different secondary metabolites was observed between elaioplasts and chromoplasts. A total of 42 and 40 core enzymes involved in secondary metabolism were identified in elaioplasts and chromoplasts, respectively (Supplementary Table S2). The MEP pathway provides substrates to synthesize terpenes, tocopherols, and carotenoids (Fig. 6). In this study, an entire set of enzymes responsible for the MEP pathway were found in both elaioplasts and chromoplasts, including DXS, DXR, CMK, MCT, MDS, HDS, and HDR. Terpenoids are specifically accumulated in elaioplasts. We found seven proteins involved in terpenoid biosynthesis, such as myrcene synthase, 1,8-cineole synthase, germacrene synthase, and limonene synthase which accounted for more than 16.7% of total proteins involved in secondary metabolism (Supplementary Table S3). By contrast, only limonene synthase (accounting for 2.5%) was identified in chromoplasts. With regard to carotenoid metabolism, ten enzymes (accounting for 25%) involved in carotenoid synthesis were identified in the chromopasts, including the rate limiting enzyme phytoene synthase, ζ-carotene desaturase (ZDS), phytoene desaturase, lycopene β-cyclase, carotenoid isomerase (CRTISO), and β-carotene hydroxylase. By contrast, only CRTISO and ZDS were identified in elaioplasts. The synthesis of volatile apocarotenoids enhances dramatically during plastid development^56,57^. Consistently, CCD1 was detected in elaioplasts and chromoplasts.

The extensive formation of plastoglobules within chromoplasts of citrus flesh is the most representative feature during chromoplast biogenesis^9^, which is confirmed by the present study (Fig. 5b). Thirty plastoglobule-localized proteins have been established in Arabidopsis^58^, and in our previous chromoplast proteome analysis we identified citrus homologs for 24 of these 30 proteins^9^. In this study, we identified five additional plastoglobule-localized proteins (Supplementary Table S4). However, it remains unclear whether plastoglobules within elaioplasts have a protein composition similar to that in chromoplasts. In elaioplasts, we only identified eight proteins that are predicted to be plastoglobule-localized using the same method. They include fibrillin 1 (FBN1), FBN7, tocopherol cyclase, flavin reductase-related 1/2, PLAT/LH2-1, and diacylglycerol acyltransferase. The FBN family is required for the development of leaf and fruit, and is involved in regulation of the extension of plastoglobules^58^. In pepper, it is reported to be important for the formation of carotenoid-lipoprotein sequestration substructures and contributes to elevated levels of carotenoid accumulation^59^. Fibrillin and its homologs were identified at high abundance in elaioplasts (Table 1), suggesting a possible role in the formation of plastoglobules. Since lipid-rich colorless elaioplasts likely have specialized but unknown functions^60^, they might possess a unique plastoglobule proteome. Therefore, the further determination of plastoglobule proteomes of this colorless, non-photosynthetic plastid is necessary and meaningful.

## Conclusion

The present study reveals several important characteristics of the elaioplasts of kumquat peel. An inventory of 655 candidate proteins predicted to be localized in citrus elaioplasts was constructed based on *in silico* homology analyses, and the majority of these proteins share similar distribution patterns in Mapman functional classes between elaioplast and chromoplast. The identification of the abundant proteins associated with the MEP pathway and terpene biosynthesis in elaioplasts suggest that this biochemical process may define a basic feature of elaioplasts. In conclusion, this proteomic analysis provides a comprehensive understanding of citrus elaioplasts as a key step towards uncovering the biogenesis of the elaioplast and its terpene metabolism.

## Electronic supplementary material

Supplemental Figures 1-3

Supplementary Table S1: Characteristics of the identified citrus plastid proteins

Supplementary Table S2: The proteome profiles of ‘secondary metabolism’ class proteins in elaioplasts and chromoplasts

Supplementary Table S3: Comparative protein profiling involved in secondary metabolites in plastids

Supplementary Table S4: The core proteome of plastoglobules of elaioplasts and chromoplasts
