# Supplementary material for: A comprehensive proteomic analysis of elaioplasts from citrus fruits reveals insights into elaioplast biogenesis and function
Source: Hortic Res. 2018 Feb 7;5:6. doi: 10.1038/s41438-017-0014-x (PMC5802726; doi:10.1038/s41438-017-0014-x)
Supplement: Supplementary file 1 — Supplemental Figures 1-3 [file 41438_2017_14_MOESM1_ESM.docx]

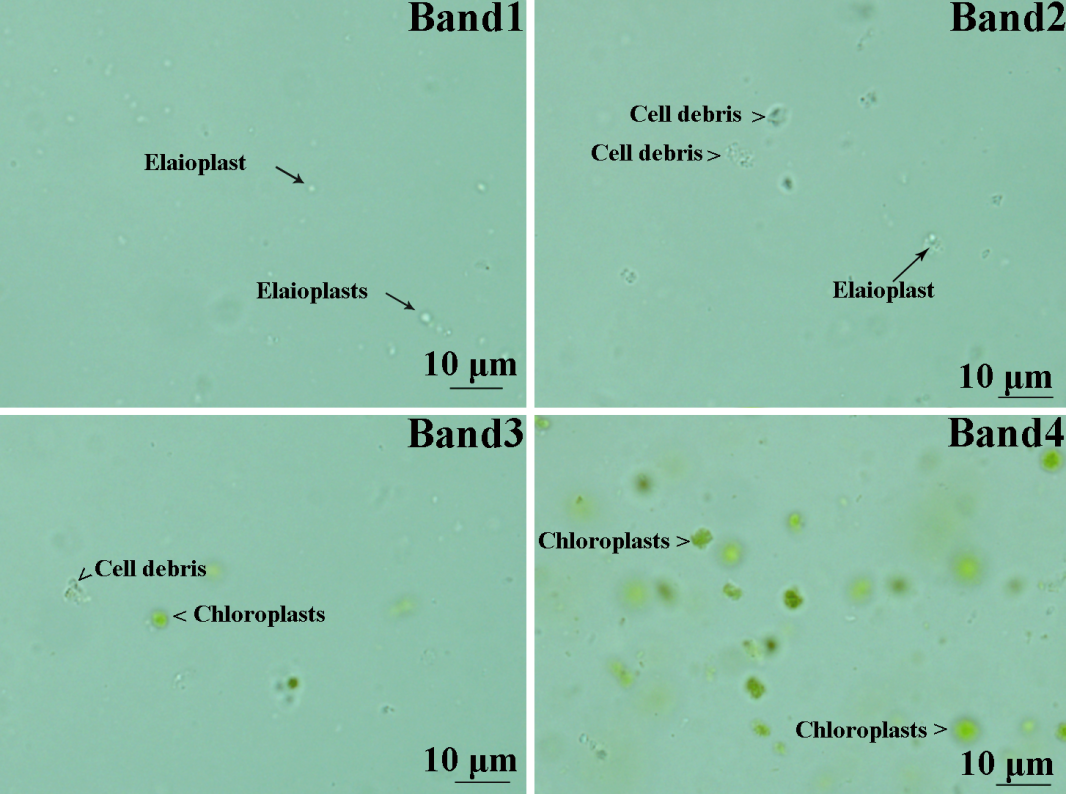


Supplementary Figure S1: A representative micrograph of pooled fractions from band 1 to band 4 isolated by a discontinuous sucrose gradient.


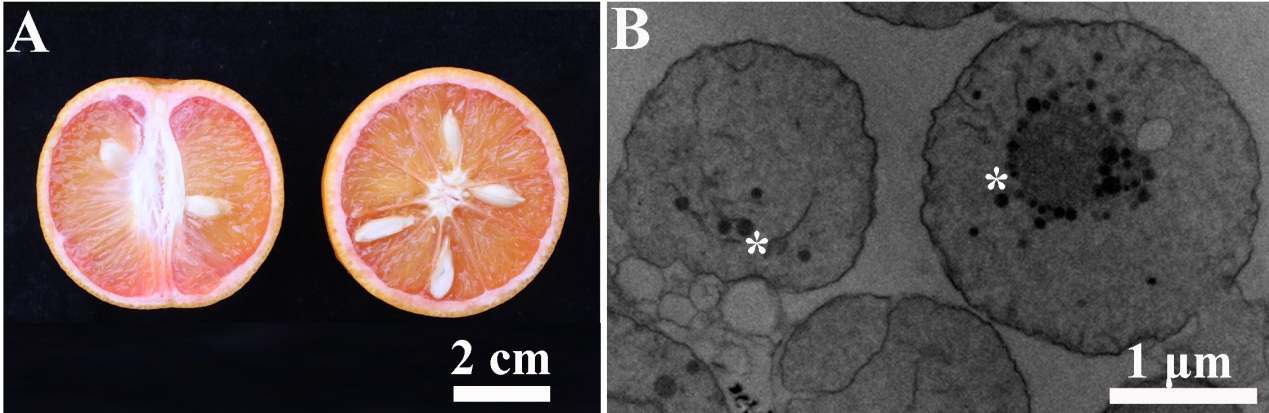


Supplementary Figure S2. Fruits of ‘Hong Anliu’ orange and isolated chromoplasts from its flesh. (A) A representative graph of sweet orange used for chromoplasts isolation. (B) A TEM image showing ultrastructural chromoplasts in the flesh of sweet orange. Asterisks indicate the plastoglobules within chromoplasts.


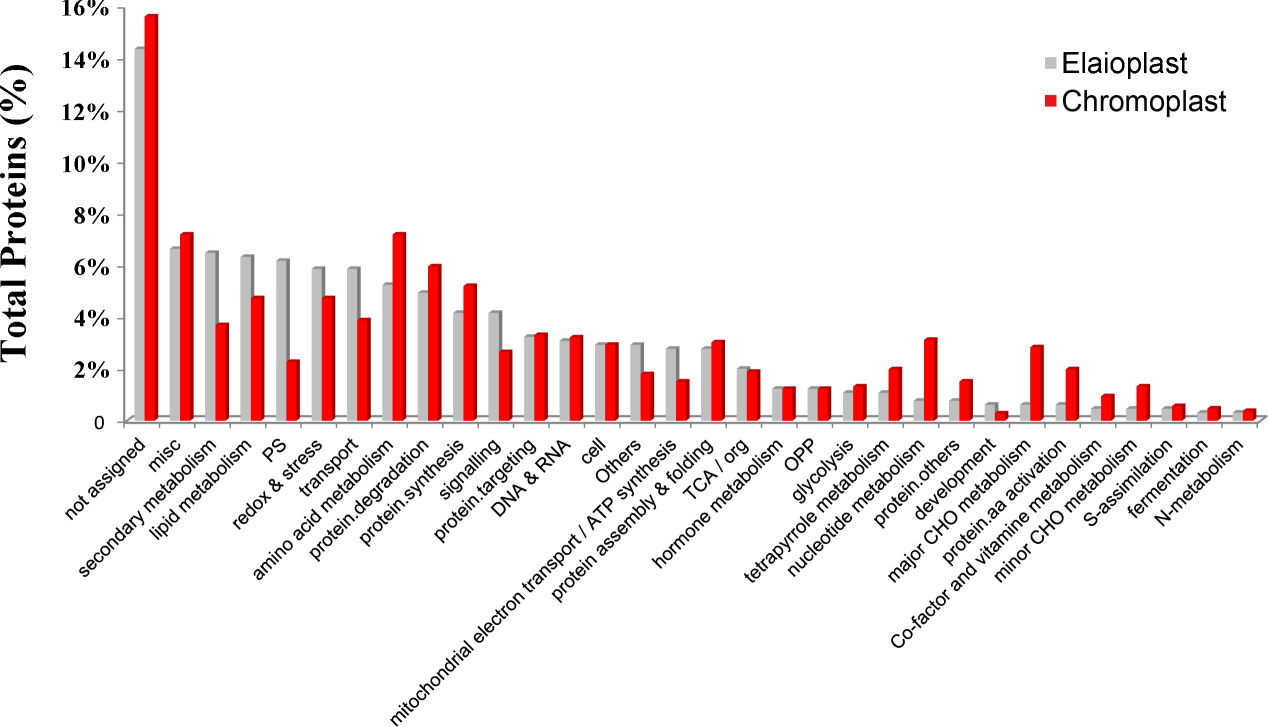


Supplementary Figure S3: Functional comparison of plastidial proteins identified from elaioplasts and chromoplasts. Proteins were classified into 32 functional categories according to MapMan (verified by PPDB, Thimm et al., 2004; <http://mapman.mpimp-golm.mpg.de/>).

***Reference***

Thimm, O., Blasing, O., Gibon, Y., Nagel, A., Meyer, S., Kruger, P., et al. (2004) MAPMAN: a user-driven tool to display genomics data sets onto diagrams of metabolic pathways and other biological processes. *Plant J.* 37: 914-939.
